# Chronic treatment with cisplatin induces chemoresistance through the TIP60-mediated Fanconi anemia and homologous recombination repair pathways

**DOI:** 10.1038/s41598-017-04223-5

**Published:** 2017-06-20

**Authors:** Wen-Pin Su, Yen-Chih Ho, Cheng-Kuei Wu, Sen-Huei Hsu, Jia-Lin Shiu, Jheng-Cheng Huang, Song-Bin Chang, Wen-Tai Chiu, Jan-Jong Hung, Tsung-Lin Liu, Wei-Sheng Wu, Pei-Yu Wu, Wu-Chou Su, Jang-Yang Chang, Hungjiun Liaw

**Affiliations:** 1 Institute of Clinical Medicine, College of Medicine, National Cheng Kung University, No.35, Xiaodong Road, Tainan 704, Taiwan; 2 Department of Internal Medicine, National Cheng Kung University Hospital, College of Medicine, National Cheng Kung University, Tainan 704, Taiwan; 3 Department of Life Sciences, National Cheng Kung University, No.1 University Road, Tainan, 701 Taiwan; 4 Department of Biomedical Engineering, National Cheng Kung University, Tainan, 701 Taiwan; 5 Department of Biotechnology and Bioindustry Science, National Cheng-Kung University, Tainan, 701 Taiwan; 6 Department of Electrical Engineering, National Cheng Kung University, Tainan, 701 Taiwan; 7 Institute of Biological Chemistry, Academia Sinica, Taipei, 11529 Taiwan; 8 National Institute of Cancer Research, National Health Research Institutes, Tainan, 704 Taiwan

## Abstract

The Fanconi anemia pathway in coordination with homologous recombination is essential to repair interstrand crosslinks (ICLs) caused by cisplatin. TIP60 belongs to the MYST family of acetyltransferases and is involved in DNA repair and regulation of gene transcription. Although the physical interaction between the TIP60 and FANCD2 proteins has been identified that is critical for ICL repair, it is still elusive whether TIP60 regulates the expression of FA and HR genes. In this study, we found that the chemoresistant nasopharyngeal carcinoma cells, derived from chronic treatment of cisplatin, show elevated expression of TIP60. Furthermore, TIP60 binds to the promoters of FANCD2 and BRCA1 by using the chromatin immunoprecipitation experiments and promote the expression of FANCD2 and BRCA1. Importantly, the depletion of TIP60 significantly reduces sister chromatid exchange, a measurement of HR efficiency. The similar results were also shown in the FNACD2-, and BRCA1-deficient cells. Additionally, these TIP60-deficient cells encounter more frequent stalled forks, as well as more DNA double-strand breaks resulting from the collapse of stalled forks. Taken together, our results suggest that TIP60 promotes the expression of FA and HR genes that are important for ICL repair and the chemoresistant phenotype under chronic treatment with cisplatin.

## Introduction

Cisplatin can cause interstrand and intrastrand crosslinks between purine bases; therefore, it is widely used to treat solid tumors^1^. However, chronic treatment with cisplatin can induce chemoresistant phenotype, which has become the major obstacle to the efficacy of the treatment. Therefore, discovering the genes underlying this chemoresistant phenotype is vital to researchers seeking to provide gene-targeted therapies aimed at treating chemoresistant cancer.

Recently, several lines of evidence have suggested that enhanced DNA damage repair pathways, including the Faconi anemia (FA), homologous recombination (HR), and post-replication repair (PRR) pathways, contribute to the chemoresistant phenotype by enhancing DNA repair ability^2–7^. Consistent with these observations, cisplatin-caused DNA lesions are majorly repaired by the FA pathway^8, 9^. Several components of HR, PRR, and nucleotide excision repair (NER) also participate in the FA pathway^8^. The FA pathway specifically resolves interstrand crosslinks during DNA replication. The FA pathway contains at least 21 genes, including 18 distinct functional complementation groups (A, B, C, D1, D2, E, F, G, I, J, L, M, N, O, P, Q, R, and S) and a few FA-associated proteins (FAAP24, MHF1, and MHF2)^10–14^. The core complex contains eight FA proteins (FANCA/B/C/E/F/G/L/M). The FANCM-FAAP24 complex recognizes the DNA lesion, recruits the FA complex, and activates ATR-mediated checkpoint signaling^12, 15^. Subsequently FANCL E3 ligase, together with UBE2T E2 conjugating enzyme, promotes the monoubiquitination of FANCD2 and FANCI^16, 17^. The monoubiquitination of FANCD2 and FANCI is the key regulatory step in the pathway, which acts as a platform to recruit several nucleases, including FAN1, SLX4, MUS81-EME1, and XPF-ERCC1, to the site of repair to initiate the incision^18–22^. The TLS DNA polymerases in the PRR pathway, such as REV1, Polκ, and Polζ participate in replicating through the DNA lesions^23–27^. The DNA double-strand breaks (DSBs) caused by the incision is subsequently repaired by HR^28–34^. Finally, NER is involved in removing the remaining adducts and in filling the gap^20, 21, 35, 36^.

TIP60 belongs to the MYST family of histone acetyltransferases^37^. It can regulate gene transcription by acetylating histone H4 at lysines 5, 8, 12, and 16, and also H2A, H2AX, and H2AZ^38–40^. Previous studies have shown that TIP60 can regulate the expression of several genes involved in the NER pathway, such as ERCC1 and APE1^41, 42^. In addition to that, TIP60 is also involved in the FA pathway by physically interacting with FANCD2 and TIP60 has been proposed as an integral factor of FA complex^43^. Interestingly, a recent study suggests that FANCD2 mediates localization of TIP60 at the ICL damage sites, where TIP60 acetylates H4K16 at the sites to block the binding of NHEJ protein, 53BP1. Therefore, the subsequent HR is recruited to repair DSBs^44^.

Despite the fact that TIP60 is an integral factor of FA pathway, it remains elusive whether TIP60 can regulate the transcription of FA and HR genes. In this study, we further identified that TIP60 can bind to the promoters of FANCD2 and BRCA1, the key regulators of the FA and HR pathways. The depletion of TIP60 expression reduces the expression of many genes in the FA, HR, TS, and TLS pathways and sensitizes cells to cisplatin. Importantly, the TIP60 deficient cells show reduced sister chromatid exchange (SCE), encounter more frequent stalled forks, and more DSBs resulting from the collapse of stalled forks. Our results suggest that TIP60 promotes the expression of FA and HR genes in a manner that is important for ICL repair and the cisplatin-resistant phenotype of cancer. Targeting TIP60 could thus be a potential therapeutic strategy for treating cisplatin-resistant cancer.

## Results

### Chronic treatment with cisplatin induces the expression of TIP60 to confer chemoresistance

**Figure 1:**
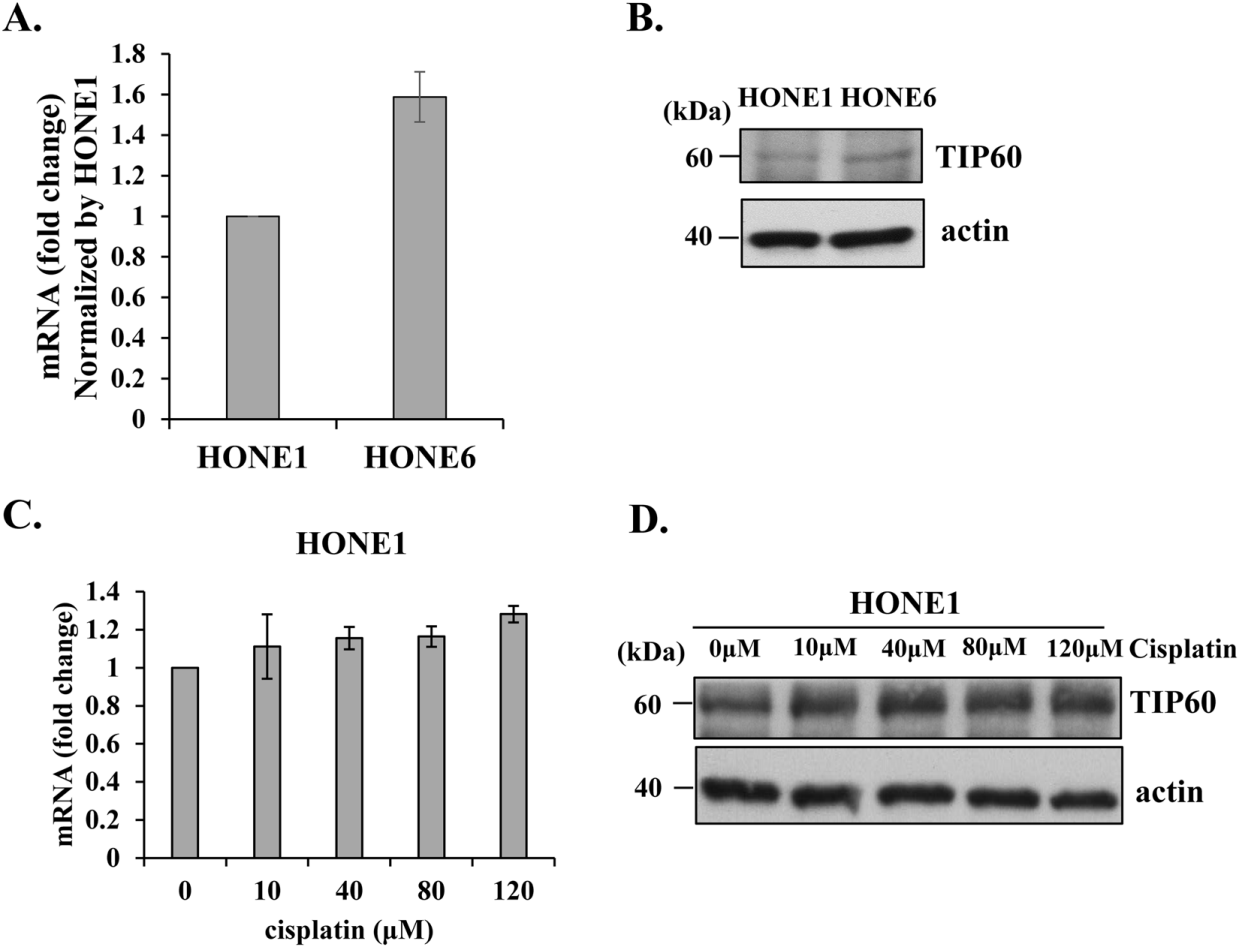
Chemoresistant HONE6 cells exhibit higher expression levels of TIP60 than cisplatin-sensitive HONE1 cells. The expression levels of TIP60 in HONE1 and HONE6 cells were determined by qRT-PCR (**A**) and Western blotting (**B**). The expression level of TIP60 in HONE6 cells was normalized by the level in HONE1 cells. (**C**) HONE1 cells were treated with various concentration of cisplatin for three hours. The expression level of TIP60 was determined by qRT-PCR and by Western blotting (**D**). Each value derived from qRT-PCR represents the mean ± standard deviation from at least three experiments. Full-length blot is presented in Supplementary Figure S4.

Previously, we have shown that chronic treatment with cisplatin can enhance the FA pathway, in coordination with the HR and PRR pathways to confer the chemoresistant phenotype in nasopharyngeal carcinoma (NPC) cells^2^. Significantly, these chemoresistant NPC cells are not only resistant to cisplatin, but also resistant to various DNA damaging agents, including mitomycin C (MMC), methylmethane sulfonate (MMS), and 4-nitroquinoline-1-oxide (4NQO)^2^. Since histone modification plays an important role in the regulation of gene expression, we hypothesize that chromatin could be remodeled to confer upon cancer cells the chemoresistant phenotype. Therefore, we used the chemoresistant NPC cell line, HONE6, which was derived from HONE1 cells by chronic treatment with low-dose cisplatin^45, 46^. As shown in Fig. 1A and B, TIP60 has a higher expression level in HONE6 cells than in HONE1 cells as judged by the mRNA and protein levels. We also found that treatment with cisplatin for three hours were sufficient to induce the expression of TIP60 in HONE1 cells (Fig. 1C and D). Our results suggest that cisplatin can induce the expression of TIP60. It is possible that TIP60 mediated histone acetylation could induce the expression of downstream genes, such as FA and HR genes to confer the chemoresistant phenotype of cells.

### Depletion of TIP60 sensitizes HONE6 cells to cisplatin

**Figure 2:**
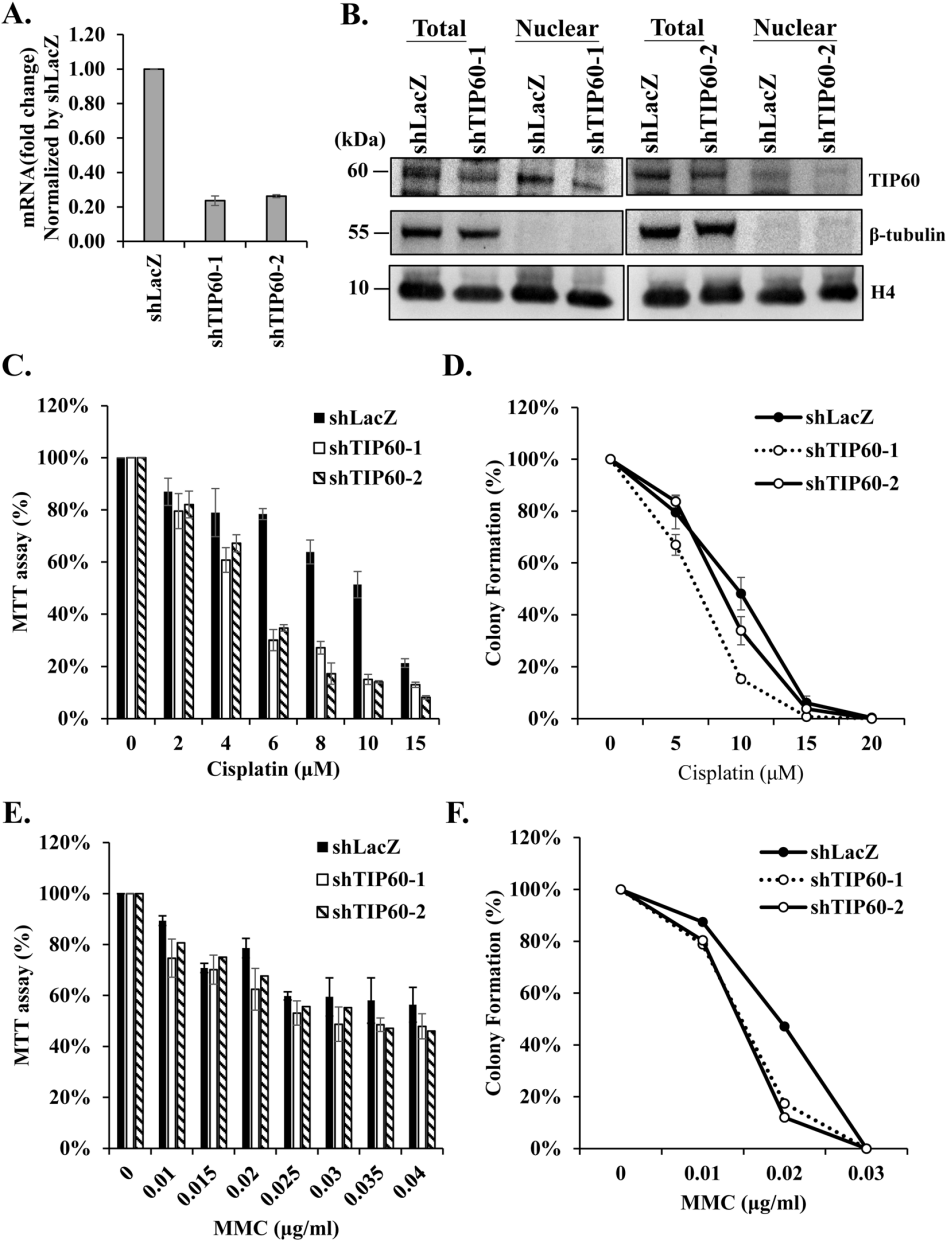
TIP60-deficient HONE6 cells are sensitive to cisplatin. (**A**) TIP60 was depleted by two specific shRNAs (shTIP60-1, and shTIP60-2) packed lentivirus in HONE6 cells. The shLacZ was used as a nontargeting control. The knockdown efficiency of TIP60 was determined by qRT-PCR and normalized by the level in the control cells. Each value represents the mean ± standard deviation from at least three experiments. (**B**) Cell lysates were subjected to Western blotting with an anti-TIP60 antibody. The total cell lysates and the nuclear fraction of cells were indicated. Full-length blot is presented in Supplementary Figure S5. (**C** and **E**) Cytotoxicity assay of TIP60-deficient cells. Cells were treated with various concentrations of cisplatin or MMC for 96 hours. Cytotoxicity was determined by MTT assay, with relative viability being normalized to colonies of no treatment control cells of each cell line. (**D** and **F**) The colony formation assay. Cells were chronically treated with cisplatin or MMC as indicated and incubated for 10 days. The resulting colonies were stained with 1% crystal violet. Relative viability was normalized to colonies of no treatment control cells of each cell line. Each value derived from the MTT assay and the colony formation assay represents the mean ± standard deviation from at least three experiments.

To determine whether TIP60 contributes to the chemoresistant phenotype, we determined the cell survival of TIP60-depleted HONE6 cells. We depleted the expression of TIP60 in HONE6 cells by treating them with shRNA packed lentivirus. Two specific shRNAs targeting TIP60, shTIP60–1 and shTIP60-2, were used. The scramble shRNA, shLacZ, was used as the control. The mRNA expression level of TIP60 was significantly reduced in the shTIP60 knockdown cells, with only 20% of the control level of TIP60 mRNA left as judged by the qRT-PCR (Fig. 2A). The protein levels of TIP60 in the shTIP60-1 and shTIP60-2 knockdown cells were also significantly reduced (Fig. 2B). We compared the growth curve between the control LacZ and the shTIP60-depleted HONE6 cells. Both of cell lines grew in the similar rate (Fig. S2), suggesting that the depletion of TIP60 had no significant effect on cell growth. Importantly, these TIP60-deficient HONE6 cells became more sensitive to cisplatin than the TIP60-proficient control cells according to the results of both MTT assay and colony formation assay (Fig. 2C and D). In addition, these TIP60-deficient HONE6 cells were also more sensitive to mitomycin (MMC) than the control cells (Fig. 2E and F). Additionally, we have compared the sensitivity of the TIP60-deficient HONE6 cells with that of HONE1 cells through the MTT assay. The HONE6 cells had 51% survival rate at 10 μM cisplatin (Fig. 2C). The depletion of TIP60 reduced the cell viability to 14% at 10 μM cisplatin (Fig. 2C). By contrast, the HONE1 cells were very sensitive to cisplatin, with only 4% of HONE1 cells survived at 10 μM cisplatin (supplementary Figure S3B). Therefore, the TIP60-deficient HONE6 cells were not as sensitive as the HONE1 cells. It could be due to the fact that the remaining amount of TIP60 proteins in the TIP60-depleted HONE6 cells could also contribute to cisplatin-resistant phenotype. However, we cannot rule out the possibility that other factors, in addition to the TIP60 gene, may also contribute to cisplatin-resistant phenotype. Nevertheless, our results suggest that TIP60 indeed contributes to the cisplatin-resistant phenotype of HONE6 cells, and that the phenotype can apply to other crosslink type DNA damage agents.

### Depletion of TIP60 results in more frequent stalled forks and elevated DSBs after treatment with cisplatin

**Figure 3:**
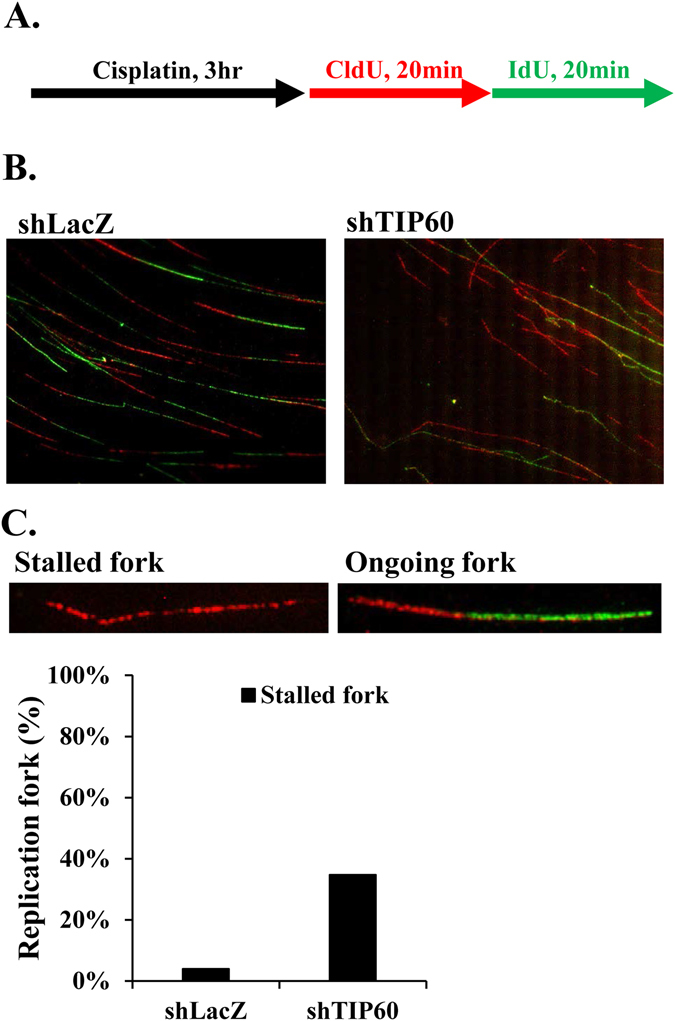
TIP60-deficient HONE6 cells show increasing frequency of stalled replication forks. (**A**) Labeling protocols for DNA fiber analysis. The HONE6 cells were treated with 10 μM cisplatin for 3 hours, followed by pulse-labelled with CldU and IdU for 20 minutes each. CldU was detected using a specific primary antibody and an Alexafluor594-labeled secondary antibody (red). IdU was detected using a specific antibody and an Alexafluor488-labeled secondary antibody (green). Representative images of replication tracks of HONE6 were shown as indicated (**B**). (**C**) Quantification of stalled forks was determined from at least 100 DNA fibers.

**Figure 4:**
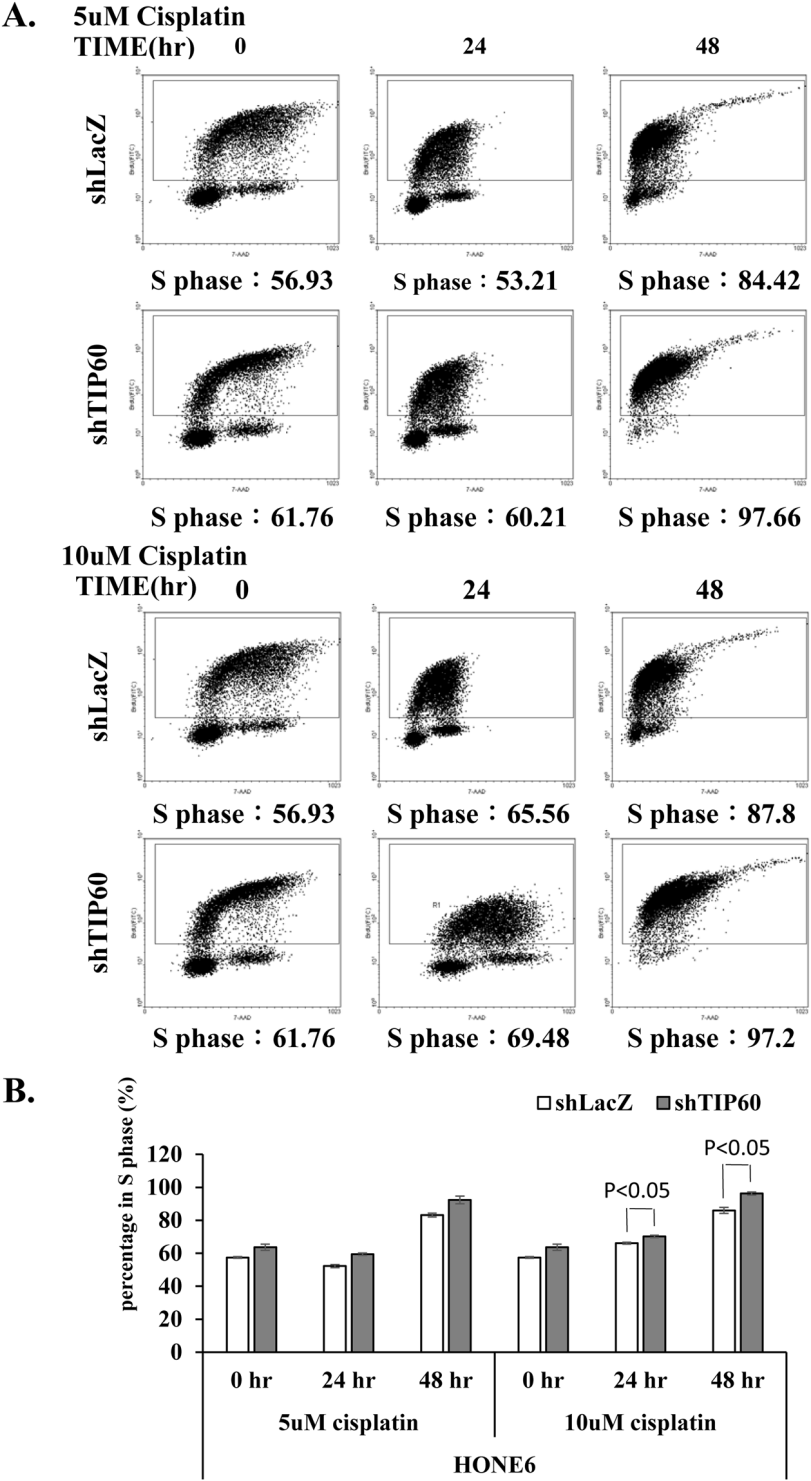
The TIP60-deficient cells accumulated significantly more cells in S-phase than the TIP60-proficient cells in 10 μM cisplatin. (**A**) Cells were chronically treated with 5 μM or 10 μM cisplatin for 24 or 48 hours. Fractions of the S phase cells were determined by the flow cytometry. (**B**) Each value represents the mean ± standard deviation from two independent experiments. The significant difference between the control LacZ and the shTIP60 HONE6 cells was indicated, with a p-value < 0.05 (Student’s t-test).

Given the fact that depletion of TIP60 sensitizes HONE6 cells to cisplatin, we investigated whether TIP60 acts in ICL repair by monitoring the progression of DNA replication in the TIP60-proficient and deficient HONE6 cells by the DNA fiber assay. Using this assay, the ongoing and stalled forks of DNA replication can be measured in a single-molecule fashion. To determine whether TIP60-deficient HONE6 cells encounter more frequent stalled forks caused by cisplatin, cells were pretreated with 10 μM cisplatin for three hours, followed by pulse-labeling with 5-chlorodeoxyuridine (CldU) for 20 minutes, and then with iododeoxyuridine (IdU) for 20 minutes (Fig. 3A). Afterward, DNA spreads were prepared and analyzed by immunofluorescence. We found that the TIP60-deficient HONE6 cells encountered more frequent stalled forks than the control cells, with a 40% frequency of stalled forks occurring in the TIP60-deficient cells in comparison to an only 5% frequency of stalled forks occurring in the TIP60-proficient control cells (Fig. 3B and C). To monitor whether the TIP60-deficient cells accumulate in the S-phase, we performed a BrdU-labelled FACS analysis. Using this analysis, the cells in the S-phase can be detected by the FITC-labelled antibodies against BrdU. As shown in Fig. 4A, chronic treatment of HONE6 cells with 5 μM or 10 μM cisplatin can cause cells to accumulate in the S-phase, with more than 80% of the cells having accumulated in the S-phase after treatment with cisplatin for 48 hours. Significantly, more TIP60-deficient cells accumulated in the S-phase, with more than 97% of those cells having accumulated in the S-phase (Fig. 4A and B). The TIP60-deficient cells accumulated significantly more cells in S-phase than the TIP60-proficient cells in 10 μM cisplatin, with a p-value of less than 0.05 (Fig. 4B). These FACS results were consistent with the results of the DNA fiber experiments, which together suggested that the TIP60-deficient cells encounter more frequent stalled forks, resulting in the accumulation of cells in the S-phase.

**Figure 5:**
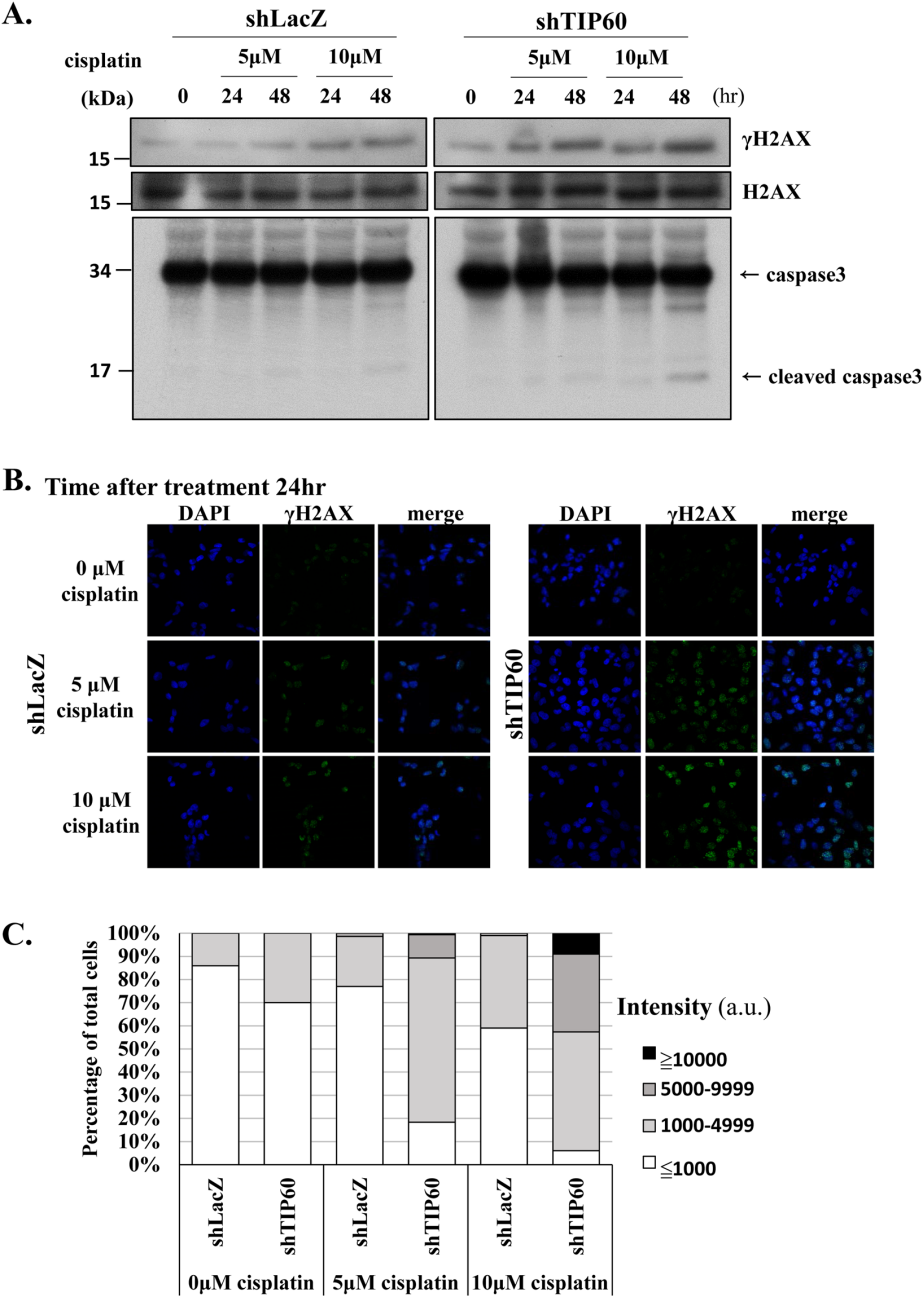
Cisplatin causes more severe DNA damage in TIP60-deficient cells. (**A**) Kinetics of DNA damage response in the control and TIP60-deficient cells. Cells were treated with 5 μM or 10 μM of cisplatin for 48 hours. Cells were harvested at the indicated time points and subjected to Western blotting with specific antibodies as indicated. The blotting of γH2AX and H2AX was from the same membrane. Full-length blot is presented in Supplementary Figure S6. (**B**) Cells were chronically treated with 5 μΜ or 10 μΜ of cisplatin for 24 or 48 hours. Cells were fixed and immunostained with γH2AX antibody. (**C**) The intensity of γH2AX in each cell was quantified using an OLYMPUS FLUOVIE software. More than 300 cells of each cell line were quantified.

To determine whether more DSBs are generated in cells due to the collapse of stalled forks, we examined the level of γH2AX and the intensity of γH2AX foci in cells using Western blotting and fluorescence confocal microscopy, respectively. Indeed, the TIP60-deficient HONE6 cells exhibited higher levels of γH2AX than the control cells after treatment with 5 μM or 10 μM of cisplatin as determined by Western blotting (Fig. 5A). The higher levels of γH2AX in the TIP60-deficient cells correlated with apoptosis as judged by the high levels of the cleaved form of caspase3 that also occurred in the cells (Fig. 5A). Consistent with the Western blotting results, the TIP60-deficient HONE6 cells also exhibited stronger intensities of γH2AX foci than the control cells after treatment with 5 μM or 10 μM of cisplatin (Fig. 5B and C). Therefore, we conclude that TIP60 plays an important role in repairing cisplatin-caused DNA damage.

### TIP60-deficient HONE6 cells show decreased the frequency of sister chromatid exchange (SCE)

**Figure 6:**
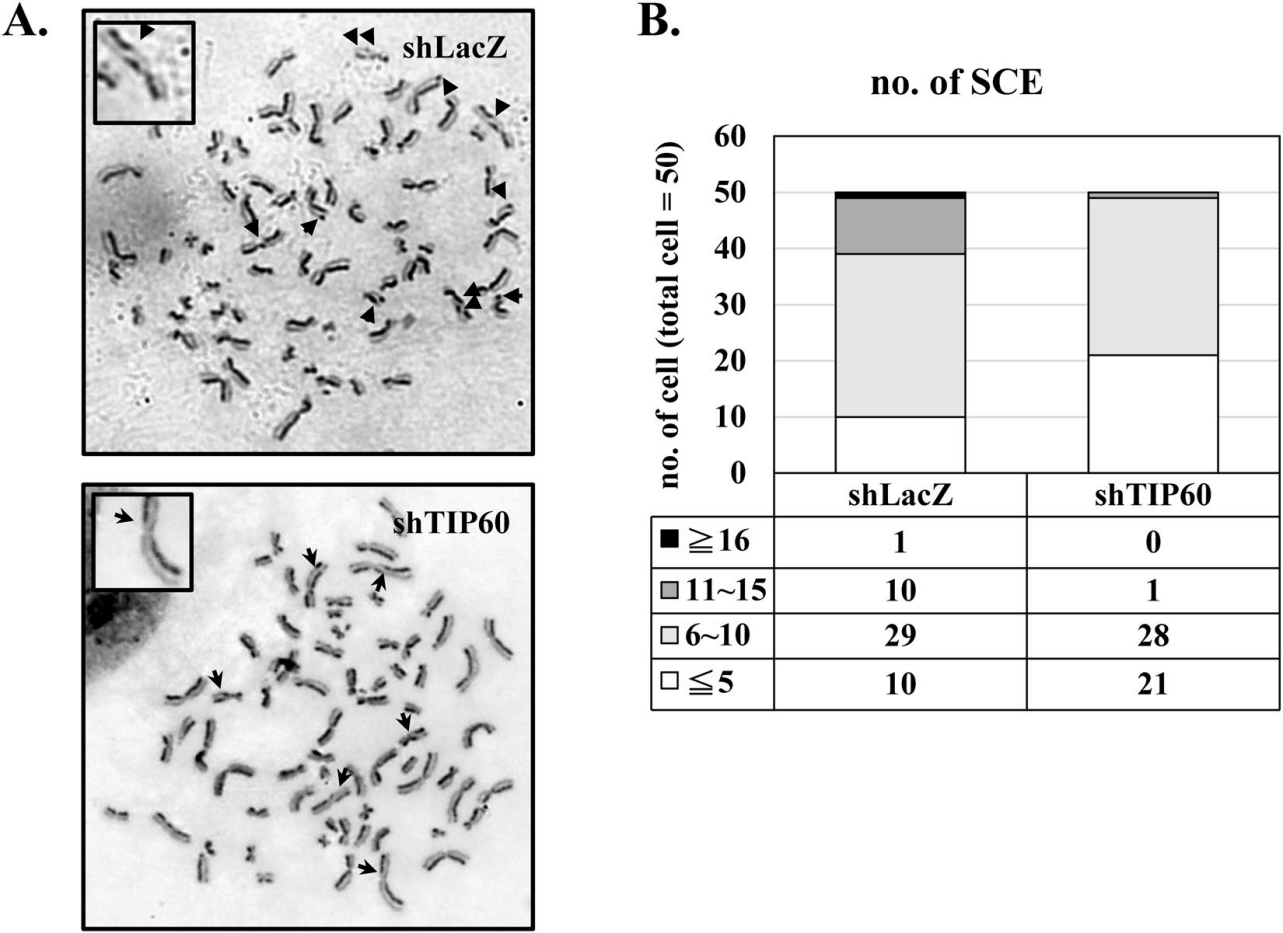
The frequency of sister chromatid exchange is significantly reduced in TIP60-deficient cells. The SCE analysis in the control and TIP60-deficient cells is shown. SCE is indicated by arrows. SCE was scored in at least 50 metaphases of each cell line.

The HONE6 cells are cisplatin-resistant cells derived from HONE1 cells by chronic treatment with cisplatin. Previously, we found that HONE6 cells show elevated frequencies of SCE, suggesting a high degree of efficiency in HR repair^2^. Since TIP60 is a histone acetyltransferase and is involved in activating transcription, we sought to examine whether TIP60 can regulate HR in HONE6 cells. Therefore, we performed the SCE analysis in the TIP60-depleted cells. As shown in Fig. 6A and B, the depletion of TIP60 significantly reduced the SCE frequency in HONE 6 cells. These results suggest that TIP60 can mediate homologous recombination, possibly through the transcription of genes involved in the HR pathway.

### The expressions of several genes in the HR, FA, and PRR pathways are reduced in HONE6-shTIP60 cells

**Figure 7:**
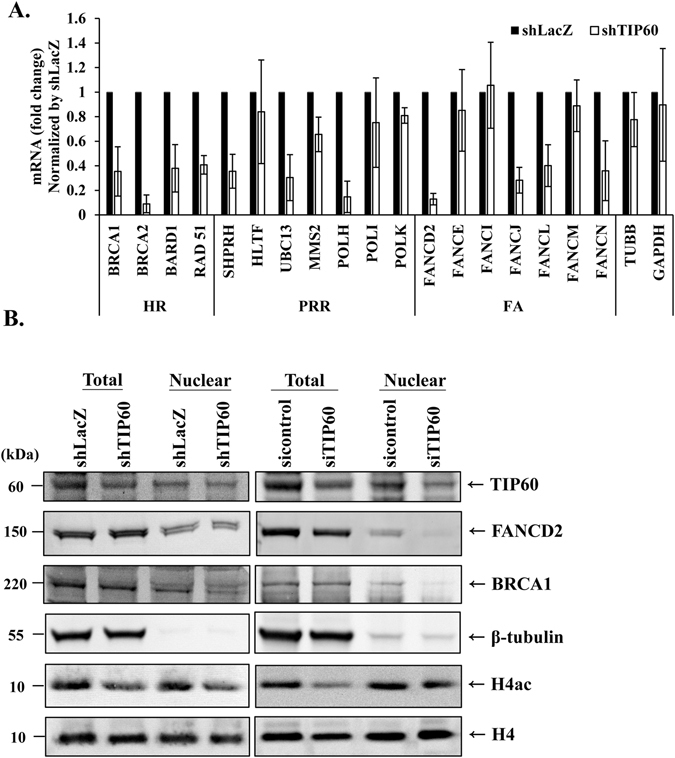
The expression of genes in the HR, TS, FA and TLS pathways are reduced in TIP60-deficient cells. (**A**) The expression levels of genes in the control and TIP60-deficient cells were determined by qRT-PCR and normalized by the levels in the control cells. Each value derived from qRT-PCR represents the mean ± standard deviation from at least three experiments. (**B**) The protein expression levels in the control and TIP60-deficient cells, including shRNA and siRNA knockdown, was determined by Western blotting with specific antibodies as indicated. Total proteins and the nuclear fraction were indicated. Full-length blot is presented in Supplementary Figure S7.

Since TIP60 can regulate HR, we tested whether TIP60 can regulate the expressions of genes in the HR pathway. Given that the FA and PRR pathways are also enhanced in the cisplatin resistant HONE6 cells^2^, genes in the FA and PRR pathways were also included. We analyzed expression levels of several genes in the FA, HR, and PRR pathways in the TIP60-deficient HONE6 cells by qRT-PCR. The HONE6 cells (shLacZ) were used as the control. As shown in Fig. 7A, we found the mRNA levels of several genes in the FA, HR, and PRR pathways were significantly reduced in the TIP60-deficient cells in comparison with the control cells. These genes include BRCA1, BRCA2, BARD1, RAD51, FANCD2, FANCJ, FANCL, FANCN, POLH, SHPRH, and UBC13. In contrast, the expression levels of some genes involved in the PRR and FA pathways, and the house keeping genes GAPDH and TUBB in the TIP60-depleted HONE6 cells were slightly reduced, but the reduction was not significant. In addition, we also examined the protein levels in the TIP60-depleted HONE6 cells. We found that the levels of TIP60 and H4 acetylation (H4ac) were significantly reduced (Fig. 7B, left panel). The results suggested that TIP60 acetylates histone H4, which is consistent with the previous publications^47^. The levels of BRCA1 is slightly reduced in the total lysates, but more significant reduction was detected in the nuclear fraction (Fig. 7B, left panel). In addition to shRNA mediated TIP60 knockdown, we also depleted the expression of TIP60 by siRNA. Similar to the results of shRNA, TIP60 was significantly reduced by the TIP60 specific siRNA (Fig. 7B). the levels of BRCA1, FANCD2, and H4ac were significantly reduced in the nuclear fraction (Fig. 7B, right panel). Based on the results of both shRNA and siRNA mediated TIP60 knockdown, we conclude that TIP60 can regulate the transcription of genes involved in the FA, HR, and PRR pathways.

### TIP60 binds to the promoter of BRCA1 and FANCD2

**Figure 8:**
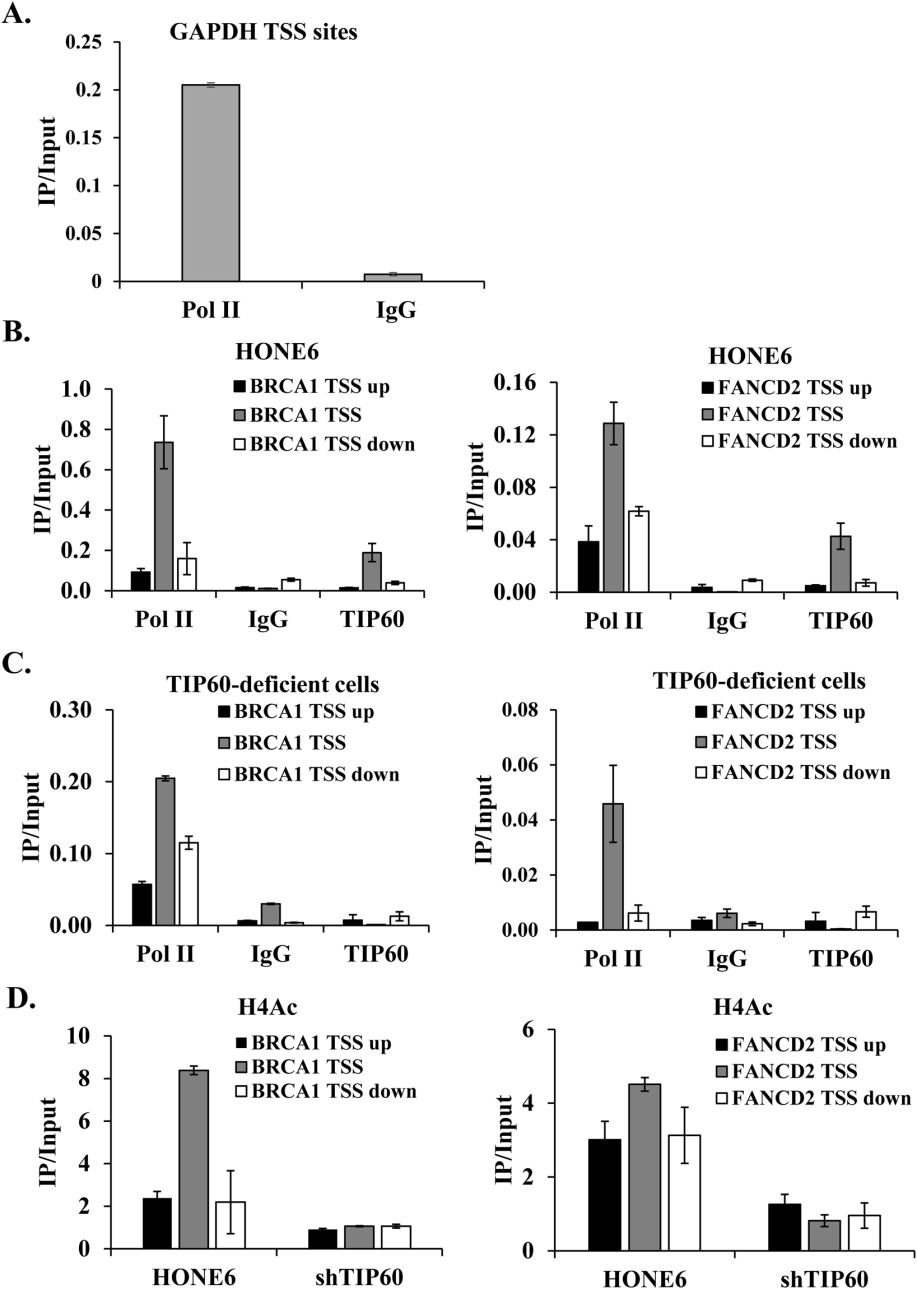
TIP60 binds to the promoter of BRCA1 and FANCD2. The ChIP experiments were performed in HONE6 and TIP60-deficient (shTIP60) cells with anti-RNA Pol II (Pol II, positive control), mouse IgG (negative control), anti-TIP60 and anti-H4ac antibodies. The immunoprecipitated DNA fragments were quantified with qPCR with specific primers located at the transcription start site (TSS) of GAPDH, and at the TSS sites and at the 2 kb sites upstream (TSS up) or downstream (TSS down) from the TSS sites of BRCA1 and FANCD2. The protein enrichment at the TSS, TSS up, and TSS down sites were determined by the amount of pulldown DNA fragments relative to the amount of the input DNA. (**A**) RNA Pol II was enriched at the TSS sites of GAPDH. (**B**) TIP60 was enriched at the TSS sites of BRCA1 and FANCD2. (**C**) the enrichment of TIP60 was dramatically reduced in the TIP60-depleted cells. (**D**) H4ac was enriched at the TSS sites of BRCA1 and FANCD2. Depletion of TIP60 dramatically reduces the enrichment of H4ac at these sites. Each value represents the mean ± standard deviation from at least three experiments.

Since TIP60 is required for the expressions of some genes involved in the HR and FA pathways, we examined whether TIP60 can bind to the promoters of BRCA1 and FANCD2 by using the chromatin immunoprecipitation (ChIP) experiments. In this experiment, the ChIP-grade antibody against TIP60 and specific primers located at the transcription start site (TSS) of BRCA1 or FANCD2 were used to test the enrichment of TIP60 at the promoter. Additionally, we also designed primers targeting 2 kb sites upstream (TSS up) or downstream (TSS down) from the TSS sites of BRCA1 and FANCD2. Since a previous study has shown that RNA polymerase II can bind to the promoter of GAPDH, the binding of RNA polymerase II at the promoter of GAPDH was used as the positive control. The nonspecific mouse IgG antibody was also included as a negative control. The amount of DNA pulled down in the ChIP reaction was quantified by qRT-PCR. These Ct values were converted to relative concentrations using a standard curve method. To represent the percentage of DNA being pulled down, the resulting IP values were normalized by the concentration of input DNA. As shown in Fig. 8A, RNA polymerase II was enriched at the promoter of GAPDH, whereas the negative control showed a trace amount of DNA being pulled down. TIP60 was enriched at the TSS sites of BRCA1 and FANCD2, but significantly reduced at the 2 kb sites upstream or downstream from the TSS sites of BRCA1 and FANCD2 (Fig. 8B). Our results suggest that TIP60 binds to the promoters of BRCA1 and FANCD2. Importantly, in the TIP60-deficient HONE6 cells, the enrichment of TIP60 at the TSS sites and at 2 kb sites upstream or downstream of the TSS sites of BRCA1 and FANCD2 was dramatically reduced (Fig. 8C). Our results suggest that the binding of TIP60 at the TSS sites of BRCA1 and FANCD2 was specific.

### Histone H4 is acetylated at the TSS sites of BRCA1 and FANCD2

Since TIP60 is enriched at the TSS sites of BRCA1 and FANCD2, we examined whether histone H4 acetylation (H4ac) was enriched at these sites. Specific antibody against H4ac was used in the ChIP experiments. As shown in Fig. 8D, H4ac was enriched at the TSS sites of BRCA1 and FANCD2 in the TIP60-proficient HONE6 cells, and the enrichment of H4ac reduced at the 2 kb sites upstream or downstream from the TSS sites of BRCA1 and FANCD2. The H4ac enrichment is corresponding to the enrichment of TIP60 binding at these sites (Fig. 8B). The depletion of TIP60 resulted in dramatic reductions of H4ac at these sites (Fig. 8D). Our results suggest that TIP60 indeed binds to the promoters of BRCA1 and FANCD2, where it acetylates histone H4 at these sites. As a result, it enhances to the expression of BRCA1 and FANCD2, which promote the HR and FA pathways.

## Discussion

Previous studies have discovered that TIP60 is involved in the base/nucleotide excision repair (BER/NER) pathways^41, 42^ and in ICL repair by directly interaction with FANCD2 at the ICL sites^43, 44^. The depletion of TIP60 reduces the expression of genes in the BER and NER pathways. These genes include APEX1, MPG, PNKP, SMUG1, DDB1, ERCC1, ERCC2, ERCC5, and TDG^41, 42^. However, none of those previous studies have used the ChIP experiments to specifically show that TIP60 binds to the promoters of these genes. Additionally, none of those previous studies provide SCE and DNA fiber evidence to show that TIP60 is involved in the HR and FA pathways. Despite all these findings, it has remained unclear whether TIP60 regulates the expression genes involved in the FA and HR pathways. In this study, however, we have further advanced the understanding of TIP60 by showing that chronic treatment of cancer cells with cisplatin can induce the expression of TIP60. TIP60 binds to the promoters of FANCD2 and BRCA1, the key regulators of the FA and HR pathways, to promote the expression of FANCD2 and BRCA1. The depletion of TIP60, on the other hand, reduces expression of several genes involved in the FA, HR, and PRR pathways and sensitizes cells to cisplatin. The involvement of TIP60 in the FA and HR pathways was also confirmed by the SCE analysis. Using DNA fiber technique, we further demonstrate that the TIP60 deficient cells encounter more frequent stalled forks. Taken together, our results suggest that TIP60 promotes the expression of FA and HR genes that are important for ICL repair and the chemoresistant phenotype under chronic treatment with cisplatin.

TIP60 belongs to the MYST family of histone acetyltransferases, which it acetylates histone H2A, H2AX, H2AZ, and H4^37–39, 47, 48^. It has been shown that TIP60 can activate transcription by binding to transcription factors^37^. These transcription factors include p53, E2F, Myc, CEBPA, HIF1A, and Fe65^49–56^. Therefore, TIP60 collaborates with transcription factors to activate transcription of genes involved in the cell cycle arrest, senescence, apoptosis, and DNA repair. Additionally, TIP60 can acetylate non-histone proteins, such as p53, p21 and ATM^57–61^. The acetylation of p53, p21, and ATM plays an important role in checkpoint activation and apoptosis^57–61^. Previous studies have shown that several transcription factors, such as E2F, NF-kB, FOXM1, NrF2, and p53, are also indicated in the chemoresistant phenotype^62^. To address the question of whether these factors are modified by TIP60 that causes chemoresistant phenotype, further studies are needed to test this possibility.

Interestingly, we also demonstrate that cisplatin can induce the expression of TIP60 both at the transcription and translation levels. Given that the chemoresistant HONE6 cells also show high expression levels of TIP60, we speculate that the induction of TIP60 could be one of the early events that induce the chemoresistant phenotype by modifying the genome of cancer cells. At the transcription level, TIP60 promotes the expressions of genes in the FA, HR, and PRR pathways; therefore, TIP60 modifies the genome of HONE6 cells, causing them to acquire the chemoresistant phenotype. However, overexpression of TIP60 in cisplatin sensitive HONE1 cells did not transform HONE1 cells into cisplatin resistant cells (Figure S3B). It indicates that additional mutations were acquired to generate the cisplatin resistant phenotype, as shown in HONE6 cells. Indeed, by using Illumina Solexa sequencing of cDNA derived from HONE1 and HONE6 cells, we found there are a lot more mutations in cDNA of HONE6 than in HONE1 cells in our preliminary results. Interestingly, several genes involved in chromatin modification are mutated in HONE6. These genes include HAT1, KAT6B, HDAC6, HDAC8, HDAC11, KDM5B, KDM6B, KDM7A, and JMJD1C. We are verifying these findings currently. Nevertheless, since depletion of TIP60 can significantly reduce the chemoresistant phenotype, TIP60 indeed contributes to the chemoresistant phenotype.

Occurring of SCE is an outcome of a series of molecular events that include chromatids breaks and rejoin via homologous recombination^63^. As a result, changes in SCE is not necessarily a reflection of changed HR efficiency alone. It can also be caused through other mechanism that directs to enhanced strand breaks such as fork collapse and mutations involved in the nucleotide excision repair. On the other hand, the DR-GFP reporter system is a more accurate assessment that focus exactly on the repairing through HR. Indeed, the depletion of TIP60 by siRNA or by using the chemical pentamidine which can inhibit the acetyltransferase activity of TIP60 can reduce HR by using the DR-GFP HR assay^64^. Therefore, the current observation of SCE changes is consistent with our hypothesis that TIP60 is involved in HR which has also been testified by using the DR-GFP HR assay^64^.

In addition to role of TIP60 in regulating the expression of FA and HR genes, TIP60 is also directly involved in the FA pathway^43, 44^. The FA complex can recruit TIP60 at the ICL sites, where TIP60 can acetylate H4. The acetylation of H4 not only block the NHEJ pathway, but also promotes HR to repair DSBs caused by the incision of ICLs^44^. Combining all the results of this study together, we propose a model in which the induction of TIP60 can not only induce the expression of FA and HR genes, but also directly involved in ICL repair. Therefore, TIP60 plays a central role for ICL repair. Indeed, several small molecules have been discovered that can inhibit the TIP60 activity^64–68^. These TIP60 inhibitors can sensitize tumor cells to ionizing radiation and increased unrepaired DNA damage in cancer cells^64, 67, 68^. Therefore, TIP60 inhibitors have a great potential to treat chemoresistant cancer.

## Materials and Methods

### Cell culture

The nasopharyngeal carcinoma cell line, HONE1, was cultured in RPMI1640 supplemented with 5% FBS, 1% glutamine, and 1% penicillin/streptomycin^45, 46^. The HONE6 cell line is the cisplatin-resistant cell line which was generated in a previous study through chronic low-dose treatment of HONE1 with cisplatin containing medium (RPMI1640 supplemented with 5% FBS, 1% glutamine, 1% penicillin/ streptomycin, and increasing dose to 5 μM cisplatin)^45^.

### Colony formation assay

Approximately 10^4^ cells were seeded in 100-mm dishes in duplicate. Subsequently, the cells were chronically treated with cisplatin or mitomycin C (MMC) for 10 days. The resulting colonies (anchorage on plates) were stained with 1% crystal violet (Sigma) for 2–3 min. After being washed with water several times to remove crystal violet solution. The colonies were then scanned with a scanner (Epson precision V33). The number of colonies was counted using the GeneTools software program (Syngene).

### Cytotoxicity assay

Cytotoxicity was determined by 3-(4,5-dimethylthiazol-2yl)-2,5-diphenyltetrazolium bromide (MTT) assay. 10^5^ cells were grown in 96-well culture plates (GeneDirex) for 24 hours and then treated with various concentrations of cisplatin and MMC for another 72 hours. 0.5 mg/ml MTT was added to each culture, followed by incubation at 37 °C for 4 hours. 10% sodium dodecyl sulfate (SDS) was added to dissolve the converted dye of the released MTT. Absorbance was measured at a wavelength of 590 nm using a microplate reader. The cytotoxicity induced by each treatment was calculated as the percentage of viable cells by dividing the optical density of samples in drug-treated wells by that of the control wells.

### Growth curve measurement

3000 cells of each cell line were seeded on a 96-well plate. Cells were harvested at 0, 24, 48, and 72-hr time points and the number of cells was determined by the MTT assay. The values from each time point were normalized to the values derived from time point 0.

### qRT-PCR analysis

Total RNA from HONE1, HONE6, and TIP60-depleted HONE6 cells was isolated using Trizol reagent (Invitrogen) and was subjected to reverse transcription using the GoScript^TM^ reverse transcription system (Promega). The resulting cDNA samples were analyzed using real-time PCR analysis (ABI StepOne Plus^TM^ Real-Time PCR Systems). The real-time PCR was performed in a 20 μl reaction with 0.8 μl of 10 μM primers, 10 μl iQ^TM^ SYBR green supermix (ROCHE), and 1 μl cDNA. The primer sequences used were as follows.

TIP60: 5′-AAC CAG GAC AAC GAA GAT GAG T-3′ and 5′-ACC CAG GAA GTC CGT TCT TAG T-3′; BRCA1: 5′-AGC AGA ATG GTC AAC TGA TGA ATA-3′ and 5′-ACT GCT GCT TAT AGG TTC AGC TTT-3′; BRCA2: 5′-AAT TAG CAT GTG AGA CCA TTG AGA-3′ and 5′-CAT CAT CTG CTT GAT CCA TTT TAG-3′; BARD1: 5′-AAA TTT GAA TGG GTA AAA GCA TGT-3′ and 5′-TAA TAA GGT TGT CCT TTG GAT GGT-3′; RAD51: 5′-CAG TGA TGT CCT GGA TAA TGT AGC-3′ and 5′-TTA CCA CTG CTA CAC CAA ACT CAT-3′; SHPRH: 5′-GCC AAA GCA CTC GTT TTC TC-3′ and 5′-TTG ATT TGG GGA TCA CGT TT-3′; UBC13: 5′-CAA TGG CAG CCC CTA AAG TA-3′ and 5′-GTC TTC CAC TGC TCC GCT AC-3′; FANCD2: 5′-ATC TGC TAT GAT GAT GAA TGC TGT-3′ and 5′-AGA GCT GCT TTC TTA TCA CCA AGT-3′; FANCJ: 5′-TCT CCA CTG GAA AAG ATA AAC TCC-3′ and 5′-AGT AAT CTG AGC AAT CTG CTT GTG-3′; FANCL: 5′-ACT ATG CTT CCT GAG TGC TTC TTT-3′ and 5′-GCA TAA CAA ATT CCA CAA TCC ATA-3′; FANCN: 5′-AAA ACT TTA TAC CTG GCA CTT CG-3′ and 5′-CCA CTG CTA CTA ACT AGC CTC CTC-3′; ACTB: 5′-AGG CAT CCT CAC CCT GAA GTA-3′ and 5′-GGG ATA GCA CAG CCT GGA TAG-3′; HLTF: 5′- GTG CAA TTT GCC TGG ATT CT -3′ and 5′- TAG CAT GTG GCT GCT CAT TC -3′; MMS2: 5′- AAG GAG TAG GCG ACG GTA CA -3′ and 5′- ACG GAG GAG CTT CTG GGT AT -3′; POLH: 5′- GTT ACC AGC TCA GAA GCT AAG ACC -3′ and 5′- TTC CTG TAC TTT GAC TGG TTT GAA -3′; POLI: 5′- CAA GCA GCT TCC AGT AGA TAT TCA -3′ and 5′- ATG GGA ATA TCT TGC ATT TGT TTT -3′; POLK: 5′- AAA TTC TTC CCA ATA GAC AAG CTG -3′ and 5′- CTA GAC CCA AGG AGA TAT GAA GGA -3′; FANCE: 5′- AGA GTT ACT GTG TTG CCT TGT GAA -3′ and 5′- ATA CTT GGT CAT CAC TGT CAG CAT -3′; FANCI: 5′- CAG AAA GAG TGT TTT GGA AGG AAT -3′ and 5′- TTA AGT GTT TCA CGA GTT CTC TGC -3′; FANCM: 5′- TAT GCT TAT TGC CAG GTT GTA AGA -3′ and 5′- CGG AAC AAT AAG CTT TTC AAC TTT -3′; GAPDH: 5′- TGC TTT TAA CTC TGG TAA AGT GGA -3′ and 5′- ATT TCC ATT GAT GAC AAG CTT CC -3′; TUBB: 5′- AGG TGA TCA GTG ATG AAC ATG G -3′ and 5′- GTC TAA AGA TCT GGC CAA AAG GAC -3′.

The real-time PCR was started at 95 °C for 10 min, followed by 40 cycles at 95 °C for 15 sec, 55 °C for 30 sec, and 60 °C for 30 sec.

The Ct values from qPCR were converted to relative concentrations using a standard curve method. The expression level of β-actin (ACTB) was used as an internal control of each cell line. The expression levels of genes in each cell line were normalized by the levels of ACTB in each cell line. To compare expression levels of genes between the shLacZ control and the TIP60-depleted HONE6 cells, the resulting number derived from the TIP60-depleted HONE6 cells was normalized with the number derived from the shLacZ control cells.

### RNA interference

HEK293T was used as packaging cells to generate shRNA encoding lentiviruses. The HONE6 cells infected with lentiviruses were selected in 2 μg/ml puromycin. All shRNA reagents were obtained from the National RNAi Core Facility, Academia Sinica. Sequences targeted by shRNAs were as follows:

- shLacZ: CGC GAT CGT AAT CAC CCG AGT (TRCN0000244984);
- shKAT5(TIP60-1): TCG AAT TGT TTG GGC ACT GAT (TRCN0000020318);
- shKAT5(TIP60-2): CCT CAA TCT CAT CAA CTA CTA (TRCN0000020315).

The siRNA specifically targeting TIP60 (ON-TARGETplus SMARTpool) and the nontargeting control siRNA (ON-TARGETplus siCONTROL Nontargeting pool) were purchased from Dharmacon and transfected as the manufacturer suggested.

The depletion of TIP60 was verified by qRT-PCR and Western blotting.

### Flow cytometry

Cells were pulsed with 10 μM bromodeoxyuridine (BrdU) for one hour. Cells were then fixed and stained for BrdU and DNA content with an anti-BrdU FITC-conjugated antibody and with a 7-aminoactinomycin D (7-AAD) dye, respectively, according to the instructions of the BD Pharmingen FITC BrdU Flow Kit (BD Biosciences FITC BrdU Flow Kit 559619, 557891). All data were collected using the BD FACSCalibur software, and results were analyzed by flow cytometry (Cell Lab Quanta™ SC Flow Cytometer, Beckman Coulter).

### Western blotting

10^6^ cells were resuspended in lysis buffer [50 mM Tris (pH7.5), 150 mM NaCl, 1 mM EDTA, 0.1% Triton X-100, protease inhibitor cocktail (MD Biol)]. After sonication, cell lysates were added with Laemmli sample buffer and boiled for 5 minutes. Samples were separated on a 4–12% gradient SDS-PAGE and transferred to a PVDF membrane. Protein blots were probed using specific antibodies against TIP60 (SC-5727, Santa Cruz), β-tublin (ab21058, abcam), BRCA1 (ab167820, abcam), FANCD2 (ab2187, abcam), H4ac (#06-598, Millipore), H4K12ac (#07-595, Millipore), H4 (#06-598, Millipore), γH2AX (#05-636, Millipore), H2AX (ab11175, abcam), β-actin (#3700, Cell Signaling Technology), and caspase 3 (#9662, Cell Signaling Technology).

To make the nuclear fraction, approximately 10^7^ cells were resuspended in buffer A [10 mM HEPES (pH 7.9), 10 mM KCl, 1.5 mM MgCl_2_, 0.34 M Sucrose, 10% Glycerol, protease inhibitor cocktail (MD Biol)] and Triton X-100 was added to a final concentration of 0.1%. The nuclear fraction was separated from the cytoplasmic fraction by centrifugation at 4 °C at 1,300 × g for 5 min. after removing the supernatant, the precipitated nuclear fraction was resuspended in lysis buffer and was subjected to Western blotting as mentioned above.

All images were acquired by the GeneGenome 5 (Bio Image, Syngene) and the γH2AX/H2AX ratios were quantified using the GeneTools software program (Syngene). These Western blotting experiments were repeated at least three times and similar trends were observed.

### Sister chromatid exchange (SCE)

10^6^ cells were incubated with 9 μg/mL 5-bromodeoxyuridine (BrdU) (Sigma) for 48 hours, followed by 0.1 μg/ml colcemid (Thermo Fischer Scientific) treatment for 40 min before standard metaphase chromosome harvest^69^. Images were acquired by Nikon eclipse 80i / NIS Elements D4.20.00. For each cell line, 50 metaphases were analyzed to determine the SCE frequency.

### Immunofluorescence microscopy

Cells plated on two-well chamber slides were chronically treated with 5 μM or 10 μM cisplatin for 24 or 48 hours and then fixed with 3.5% paraformaldehyde for 15 min and permeabilized with 1% Triton X-100 for 10 min. Fixed cells were blocked with 5% FBS and stained with anti-γH2AX and Alexa-conjugated anti-mouse secondary antibodies. Images were acquired by confocal microscope Nikon C1-Si.

### Chromatin immunoprecipitation

The chromatin immunoprecipitation (ChIP) assays were performed using a ChIP assay kit (17–10085; Millipore) according to the manufacturer’s instructions. Briefly, a total of 4.0 × 10^6^ cells was fixed in 1% formaldehyde at room temperature for 10 minutes. 125 mM glycine was used to quench the unreacted formaldehyde. The cells were then washed with ice-cold PBS and lysed with cell lysis buffer to remove the cytoplasmic fraction. The chromatin fraction was resuspended in nuclear lysis buffer and then sonicated to shear DNA to about 500 bp. The immunoprecipitation was performed with 1 μg of anti-TIP60 (SC-5727, Santa Cruz), anti-H4ac (#06-598, Millipore), anti-RNA polymerase II (validated antibody for positive control included in the ChIP kit from EZ-Magna ChIP^TM^, Millipore, cat no. 17-10086) and normal mouse IgG antibodies, followed by immunoprecipitation with protein A/G magnetic beads. The immunoprecipitated DNA was reverse cross-linked with 100 μl ChIP elution buffer with 1 μl protease K (EZ-Magna ChIP^TM^, Millipore, cat no. 17-10086) at 62 °C for 2 hours, followed by incubation at 95 °C for 10 minutes. DNA was purified with a spin column (EZ-Magna ChIP^TM^, Millipore, cat no. 17-10086). Finally, the amount of DNA was analyzed by qPCR.

The qPCR was performed in a 40 μl reaction with 0.8 μl each of 10 μM primers, 0.8 μl 50 mM MgCl2, 20 μl iQTM SYBR Green Supermix (BIO-RAD iQTM SYBR Green Supermix kit), and 5 μl ChIP DNA. The PCR cycling was started at 95 °C for 3 min, followed by 40 cycles at 95 °C for 15 sec, and 55 °C for 45 sec. The primer sequences used for qPCR were as follows: TSS of BRCA1: 5′-ATT GGA TGT TCC TCT CCA TAA GAC-3′ and 5′-CAG TAA TTG CTG TAC GAA GGT CAG-3′; 2 kb upstream of TSS of BRCA1: 5′- CTT TTT AGT AGA GTC GGG GTT TCA -3′ and 5′- GTA AA A TGG ACC AAT CAA CAG GAT -3′; 2 kb downstream of TSS of BRCA1: 5′- GGT ATT TGG AAG AAC CTT TGT TTG -3′ and 5′- ACA CCT ATC GTC CCT GCT ACT CT -3′; TSS of FANCD2: 5′-GAA CCT AGG CAA ACT GAC ACA AC-3′ and 5′-GTC CTA CCC ATC GTT ATC ACT CTT-3′; 2 kb upstream of TSS of FANCD2: 5′- TGA CAG GTA TTA AAC CAA AGC TGA -3′ and 5′- CAA AGT GCT GGG ATT ATA AGT GTG -3′; 2 kb downstream of FANCD2: 5′- GTA CGT TGG GGA TAG ATA ATG GAG -3′ and 5′- AGG TGC TAA GCA AAT TTC AAC TCT -3′; GAPDH: 5′-TAC TAG CGG TTT TAC GGG CG-3′ and 5′-TCG AAC AGG AGG AGC AGA GAG CGA-3′. The ChIP data was quantified using the following formula: IP/Input, where IP and Input are the immunoprecipitated DNA and input DNA, respectively. The ChIP data was generated from at least three independent experiments. A similar trend was observed when these experiments were replicated.

### DNA fiber analysis

1.0 × 10^6^ cells were seeded in 100-mm dishes overnight, pretreated with 10 μΜ cisplatin for 3 hours, then treated with 25 μM CldU for 20 minutes, followed by 250 μM IdU for 20 minutes. CldU was detected by a specific primary antibody (Abcam) and an Alexafluor 594-labeled secondary antibody (Molecular Probes). IdU was detected by a specific antibody *(Becton Dickinson Immunocytometry Systems)* and an Alexafluor 488-labeled secondary antibody (Molecular Probes). Images were acquired by Nikon eclipse 80i / NIS Elements D4.20.00. For each cell line, at least 100 DNA fibers were analyzed to determine the progression of DNA replication.

## Electronic supplementary material

supplementary materials
