# Supplementary material for: Chronic treatment with cisplatin induces chemoresistance through the TIP60-mediated Fanconi anemia and homologous recombination repair pathways
Source: Sci Rep. 2017 Jun 20;7:3879. doi: 10.1038/s41598-017-04223-5 (PMC5478611; doi:10.1038/s41598-017-04223-5)
Supplement: Supplementary file 1 — supplementary materials [file 41598_2017_4223_MOESM1_ESM.pdf]

Chronic treatment with cisplatin induces chemoresistance through the TIP60-mediated Fanconi anemia and homologous recombination repair pathways

Wen-Pin Su[1,2,#,*], Yen-Chih Ho[3,#], Cheng-Kuei Wu[1,#], Sen-Huei Hsu[3], Jia-Lin Shiu[3], Jheng-Cheng Huang[1], Song-Bin Chang[3], Wen-Tai Chiu[4], Jan-Jong Hung[5], Tsung-Lin Liu[5], Wei-Sheng Wu[6], Pei-Yu Wu[7], Wu-Chou Su[2], Jang-Yang Chang[2,8], Hungjiun Liaw[3,*]

[1] Institute of Clinical Medicine, College of Medicine, National Cheng Kung University, No.35, Xiaodong Road, Tainan 704, Taiwan

[2] Department of Internal Medicine, National Cheng Kung University Hospital, College of Medicine, National Cheng Kung University

[3] Department of Life Sciences, National Cheng Kung University, No.1 University Road, Tainan City 701, Taiwan

[4] Department of Biomedical Engineering, National Cheng Kung University

[5] Department of Biotechnology and Bioindustry Science, National Cheng-Kung University

[6] Department of Electrical Engineering, National Cheng Kung University

[7] Institute of Biological Chemistry, Academia Sinica

[8] National Institute of Cancer Research, National Health Research Institutes, Tainan, Taiwan

[#] These authors contributed equally to this work.

[*] Corresponding authors

Address correspondence to: Hungjiun Liaw, Department of Life Sciences, National Cheng Kung University, No. 1 University Road, Tainan 701, Taiwan

Tel) 886-6-275-7575 ext 65535, Fax) 886-6-274-2583, email) liawh@mail.ncku.edu.tw

or to: Wen-Pin Su, Institute of Clinical Medicine, College of Medicine, National Cheng Kung University, No. 35, Xiao-dong Rd, Tainan 704, Taiwan; Tel) 886-6-235-3535 ext 4252, Fax) 886-6-275-8781, email) wpsu@mail.ncku.edu.tw

Supplementary Materials

Supplementary Figures S1-S8

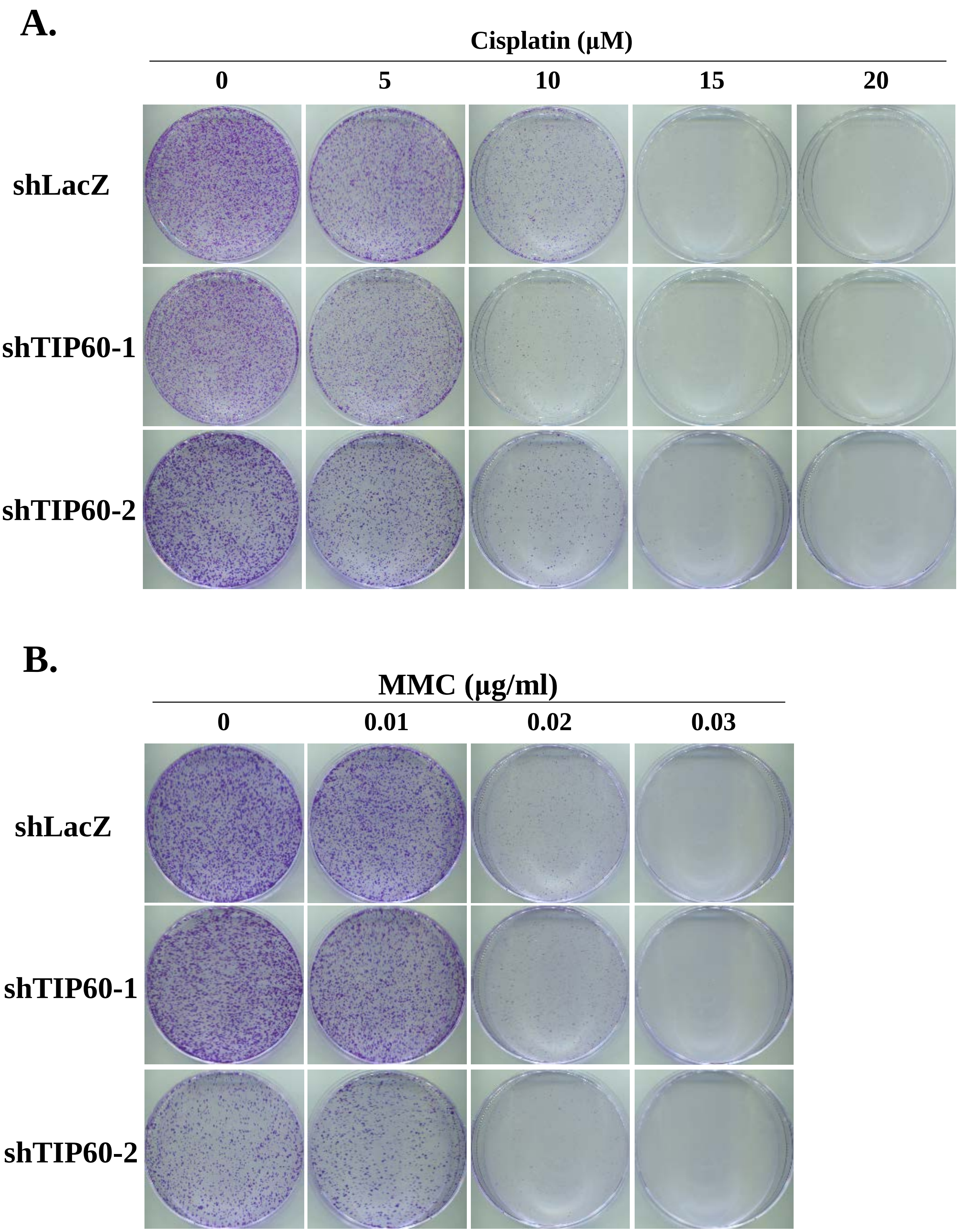

Supplementary Figure S1. The colony formation assay. The shLacZ and shTIP60 cells were chronically treated with cisplatin (A) or MMC (B) as indicated and incubated for 10 days. The resulting colonies were stained with 1% crystal violet.

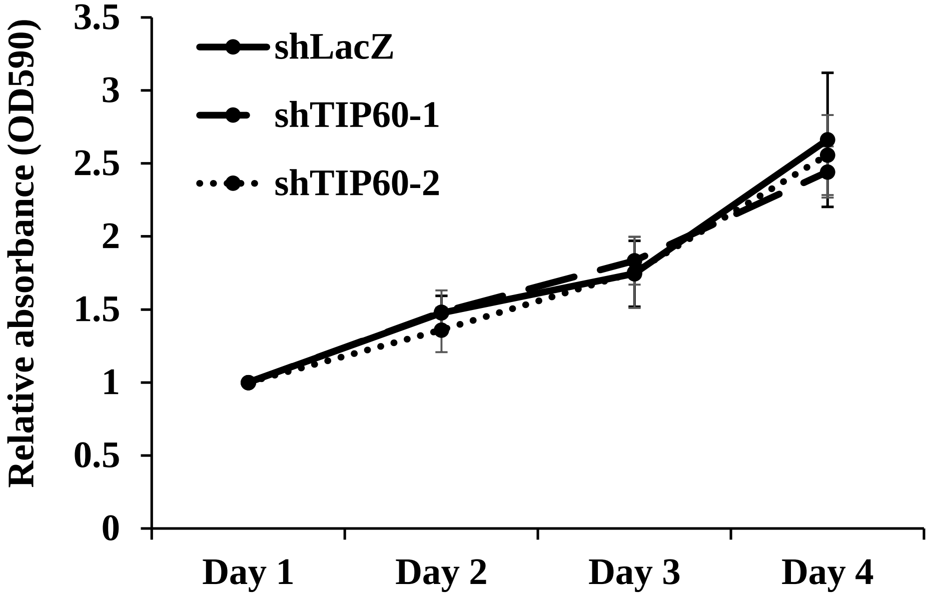

Supplementary Figure S2. The cell growth curve of the control shLacZ, shTIP60-1, and shTIP60-2 HONE6 cells. The cell growth was determined by the MTT assay.

A.

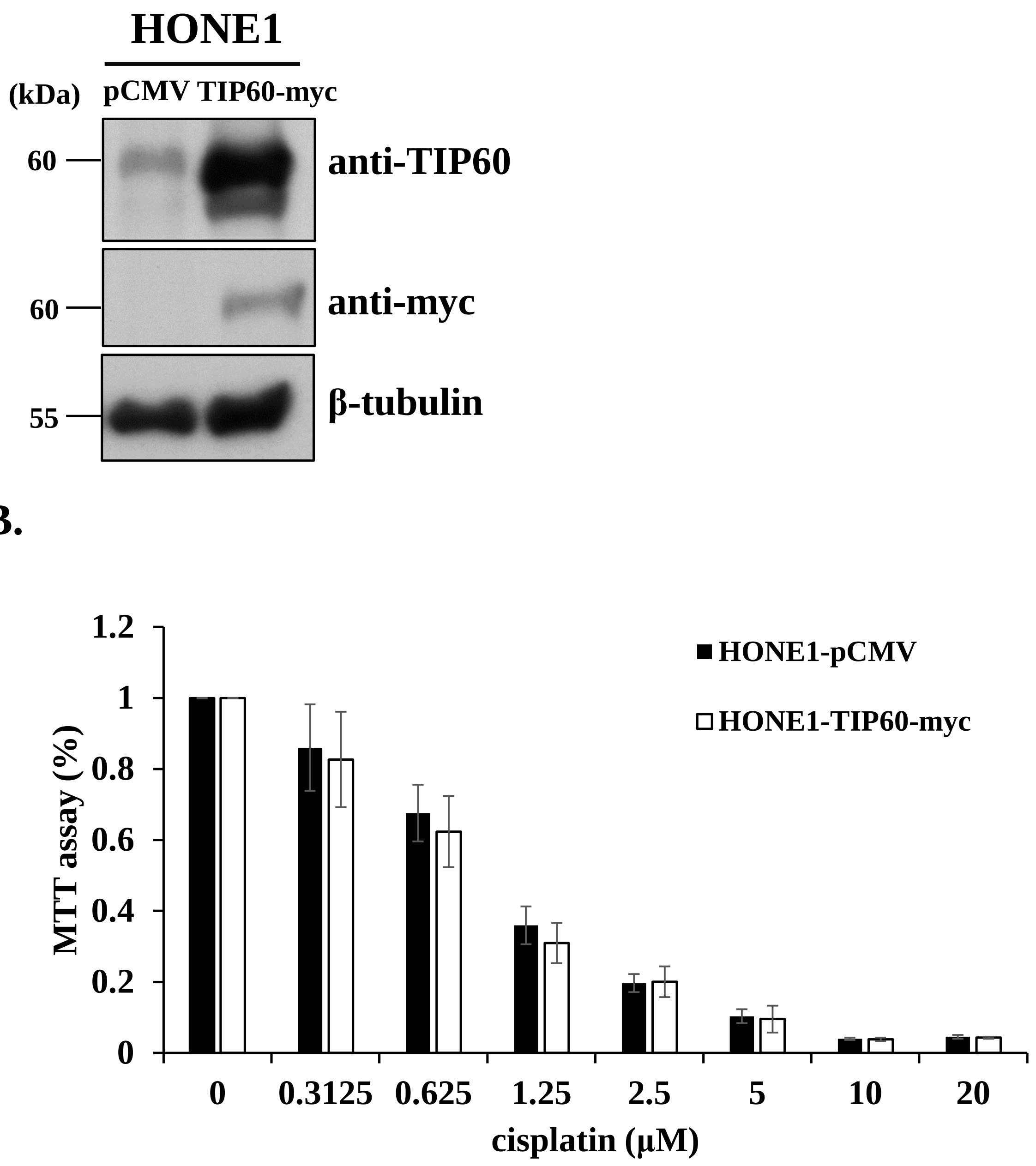

Supplementary Figure S3. The MTT assay of the control (pCMV) and the TIP60-overexpressed (TIP60-myc) HONE1 cells. (A) The western blotting of the control (pCMV) and the TIP60-overexpressed (TIP60-myc) HONE1 cells with specific antibodies was shown as indicated. The HONE1 cells were transfected with the control vector (pCMV) or vectors containing the TIP60-myc fusion gene (TIP60-myc). (B) The cytotoxicity assay of the TIP60-overexpressed HONE1 cells. Cells were treated with various concentration of cisplatin for 96 hours. Cytotoxicity was determined by the MTT assay with relative viability being normalized to values of no treatment control cells of each cell line. Full-length blot is presented in Supplementary Figure S8.

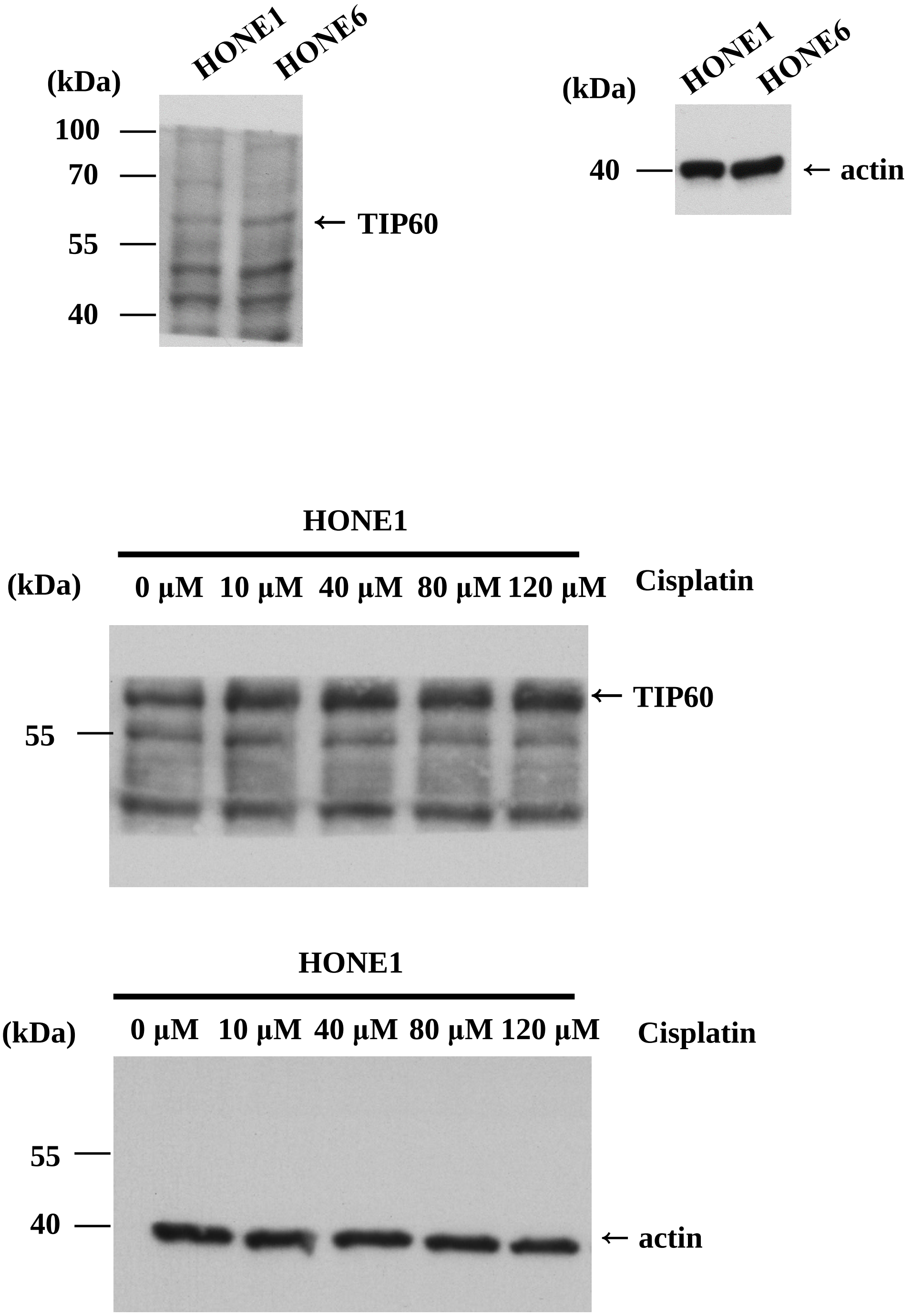

Supplementary Figure S4. Full Blots for Figure1. The protein marker was from Thermo Scientific PageRuler Prestained Protein Ladder (Thermo) #26616LCS. These images were acquired by the X-ray film.

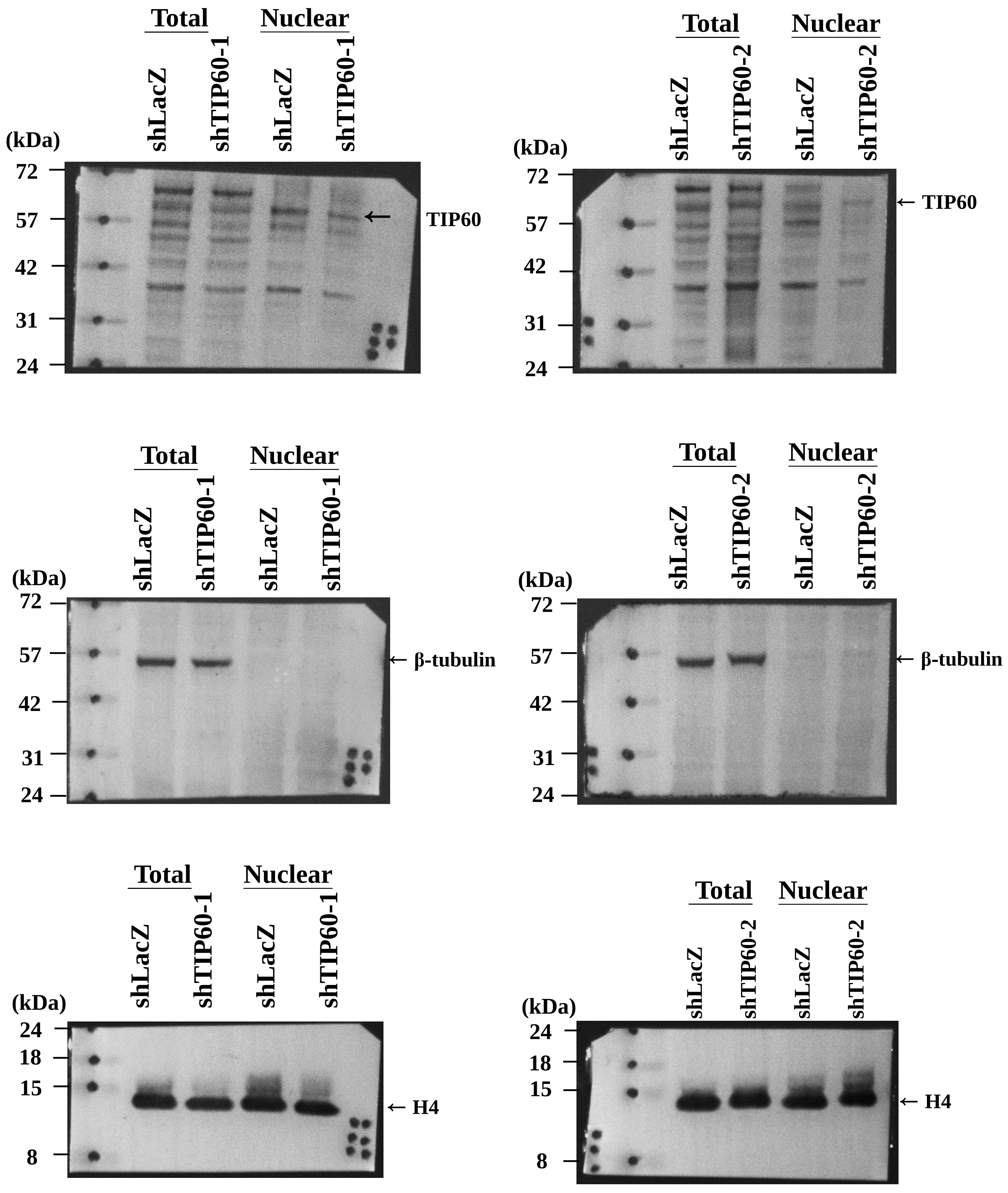

Supplementary Figure S5. Full Blots for Figure 2. The protein marker was from BLUeye Prestained Protein Ladder (GeneDirex) Cat No. PM007-0500. These images were acquired by the GeneGenome 5 (Bio Image, Syngene)

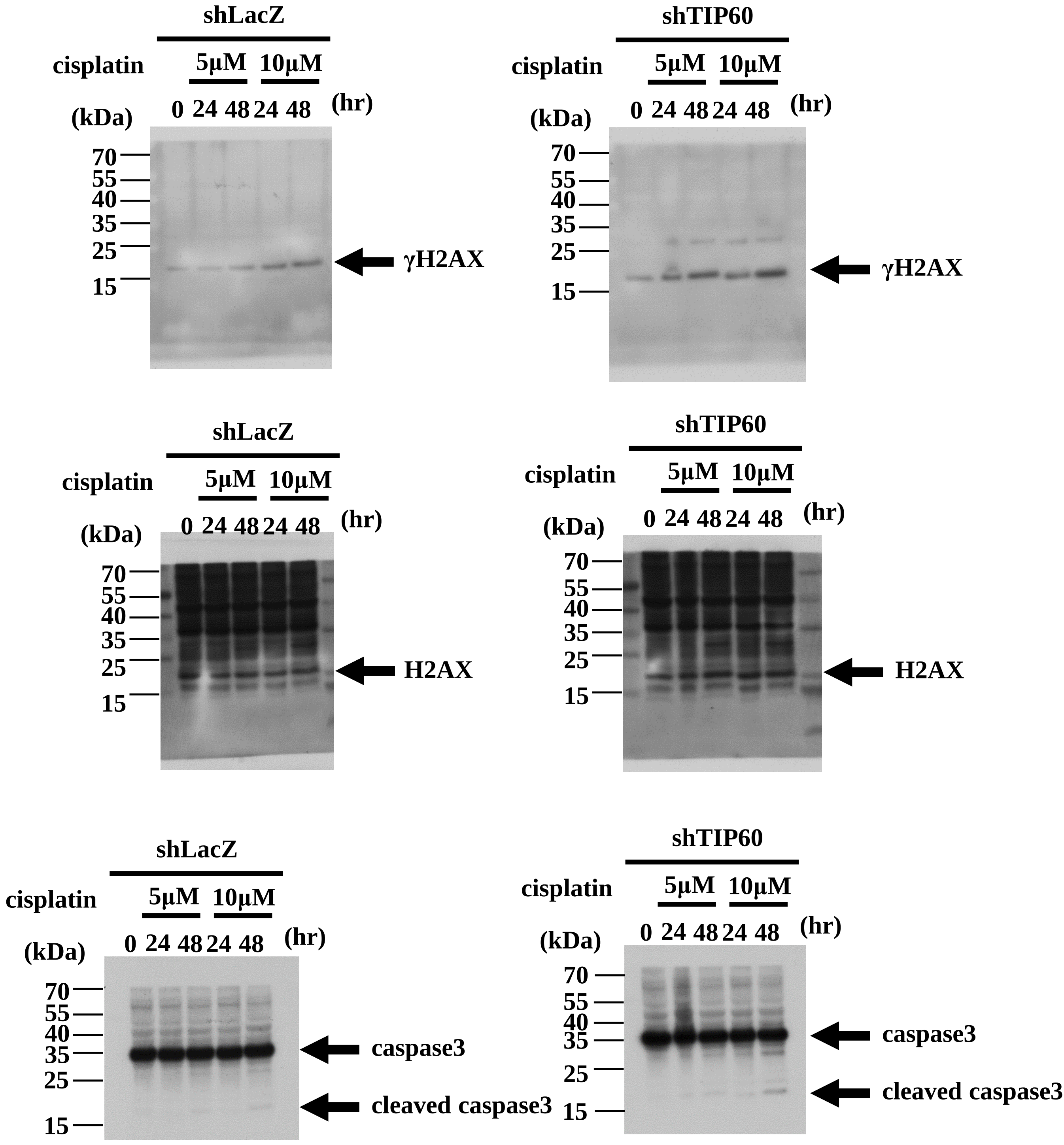

Supplementary Figure S6. Full Blots for Figure 5. The protein marker was from Thermo Scientific PageRuler Prestained Protein Ladder (Thermo) #26616LCS. These images were acquired by the X-ray film.

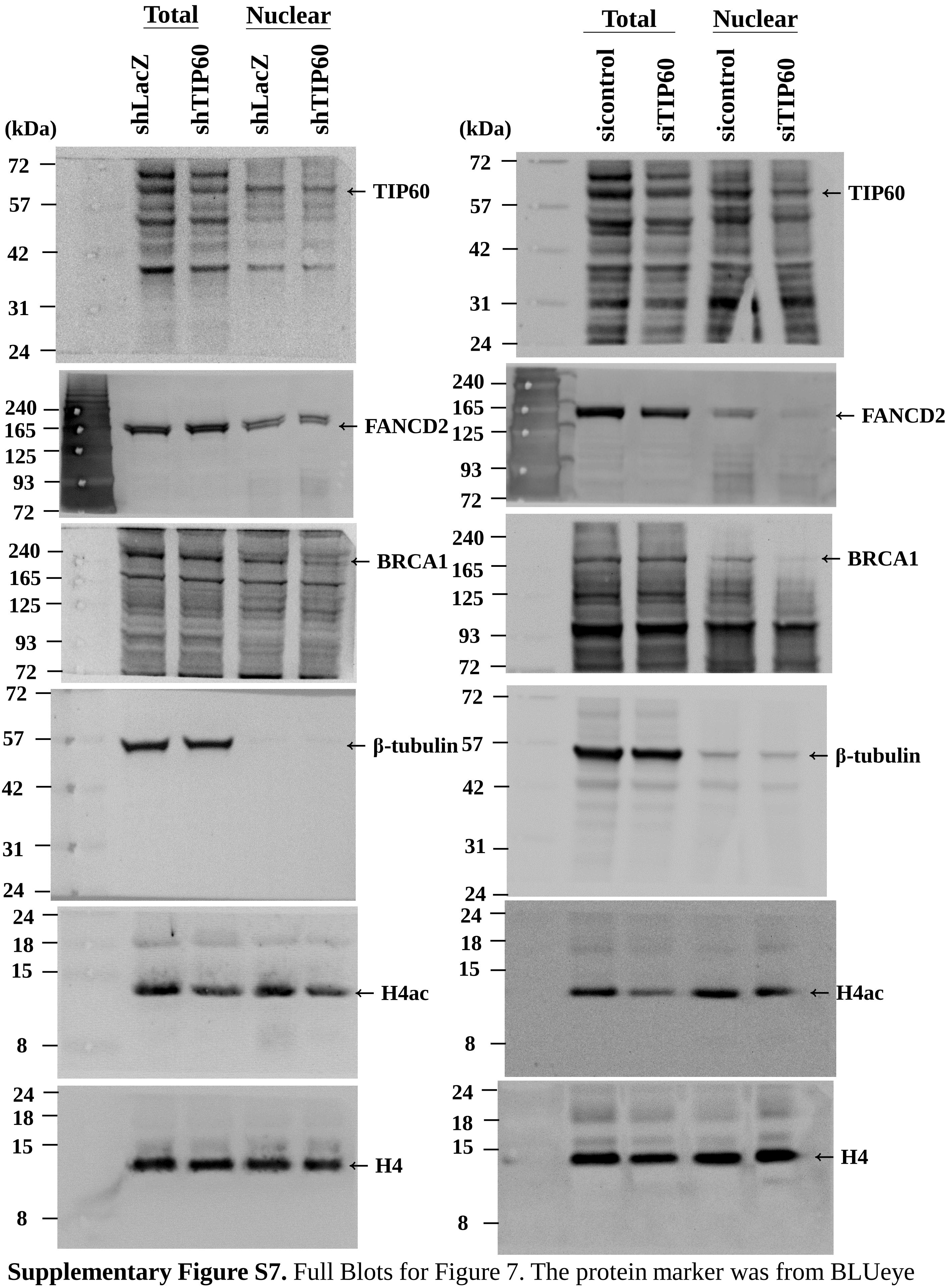

Supplementary Figure S7. Full Blots for Figure 7. The protein marker was from BLUeye Prestained Protein Ladder (GeneDirex) Cat No. PM007-0500. These images were acquired by the GeneGenome 5 (Bio Image, Syngene)

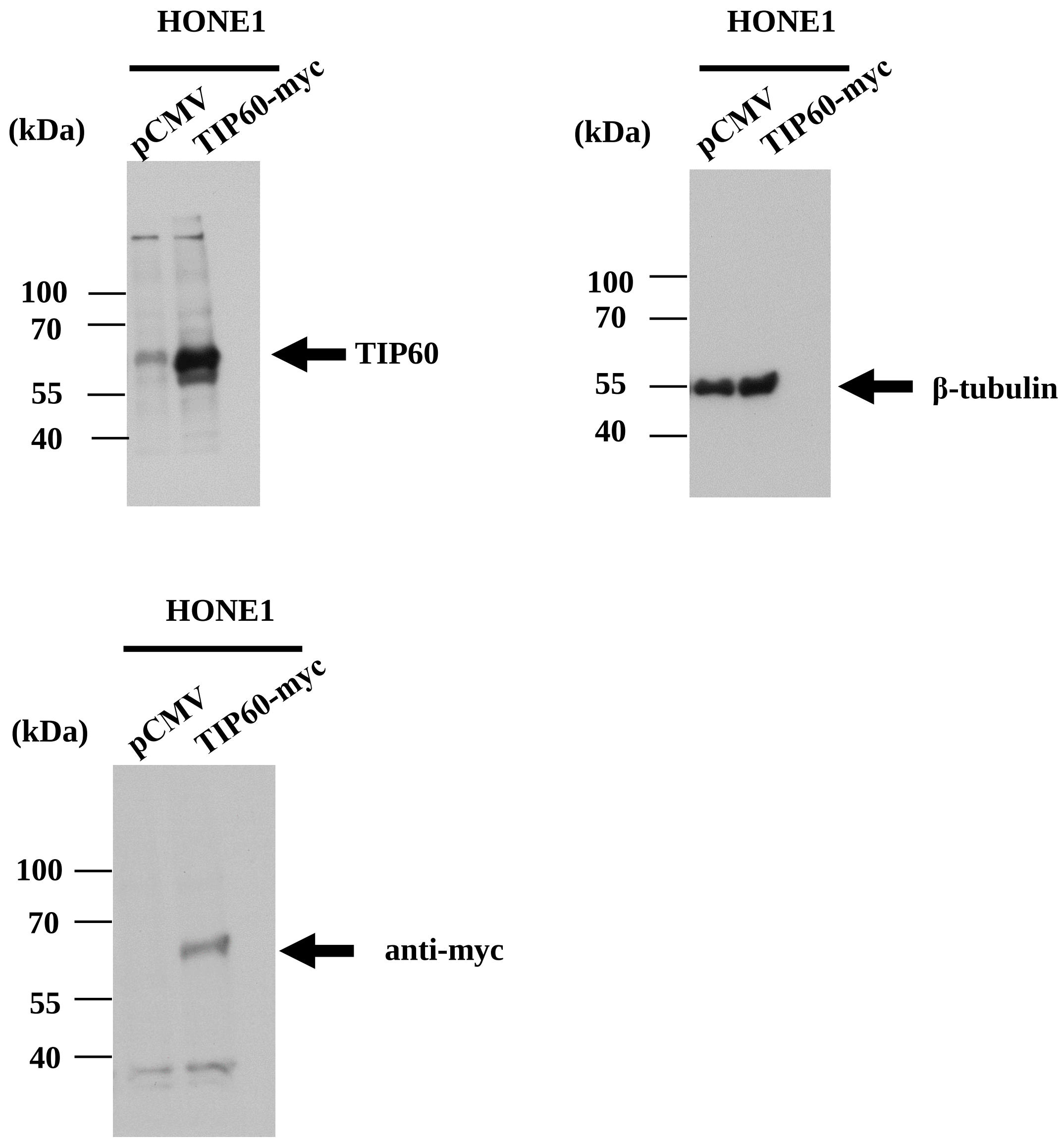

Supplementary Figure S8. Full Blots for Supplementary Figure S3. The protein marker was from Thermo Scientific PageRuler Prestained Protein Ladder (Thermo) #26616LCS. These images were acquired by the X-ray film.
